# Cognitive impairment as a central cholinergic deficit in patients with Myasthenia Gravis

**DOI:** 10.1016/j.bbacli.2015.04.003

**Published:** 2015-04-23

**Authors:** Antonia Kaltsatou, Dimitris Fotiou, Dimitris Tsiptsios, Anastasios Orologas

**Affiliations:** Neurology Clinic of AHEPA Hospital, Neuroscience Division, Medicine School, Aristotle University of Thessaloniki, Greece

**Keywords:** Myasthenia Gravis, Pupillometry, Autonomic nervous system, Cholinergic deficit, Cognitive dysfunction

## Abstract

**Background:**

The purpose of this study was to investigate with neurophysiological and neuropsychological methods such as pupillometry, cognitive test and Hamilton Depression Rating Scale (HAM-D) the hypothesis of Central Nervous System (CNS) cholinergic involvement in patients with Myasthenia Gravis (MG).

**Methods:**

Thirty-two patients (32) with MG and a mean age of 51.1 ± 17.2 volunteered to participate in this investigation, while thirty-three (33) healthy subjects with a mean age of 50.2 ± 14.8 served as controls. All subjects underwent pupillometric measurements and performed the Wechsler Memory Scale (WMS) and HAM-D. The pupillometric indices studied were: 1) latency for the onset of constriction (T1), 2) maximum constriction velocity (VCmax) and 3) maximum constriction acceleration (ACmax).

**Results:**

T1 was found significantly increased by 21.7% (p < 0.05) in MG patients as compared to healthy subjects. Conversely, VCmax and ACmax were significantly decreased in MG patients by 33.3% (p < 0.05) and 43.5% (p < 0.05) respectively, as opposed to healthy subjects. Additionally, MG patients showed significantly decreased score in WMS by 41.6% (p < 0.05) as compared to healthy controls. No significant difference was found for HAM-D between the two groups.

**Conclusions:**

VCmax and ACmax are governed mainly by the action of the Parasympathetic Nervous System, through acetylcholine. The results of this study demonstrate that the CNS may be affected in MG and support the hypothesis that MG has central cholinergic effects manifested by cognitive dysfunction.

## Highlights

• Myasthenia Gravis has central cholinergic deficits manifested by cognitive dysfunction.
• The hypothesis of Central Nervous System cholinergic involvement was examined with pupillometry and cognitive tests.
• Central Cholinergic System and/or the iris sphincter smooth muscle might be affected in Myasthenia Gravis.

## 1 Introduction

Myasthenia gravis (MG) is an autoimmune disease with increasing incidence per year. The prevalence rate of MG range from 15 to 179 cases per million worldwide [1] and Greece is reported to be among the countries with the highest one [2]. MG is widely considered to be a disorder of the neuromuscular junction, while the Central Nervous System (CNS), cardiac and smooth muscles remain unaffected [3]. MG is characterized by fluctuating fatigue and muscle weakness [4], which symptoms in some patients are limited to ocular muscles, while in others, there is an expansion to the rest of the muscles. Abnormal fatigability of voluntary muscles results from a reduced number of functional nicotinic acetylcholine receptors (AChRs) at the neuromuscular junction [5]. Nicotinic AChRs are found in the central, as well as in the peripheral nervous system, particularly in the hippocampus, hypothalamus, midbrain and cerebral cortex. It has been established that the central cholinergic system is important in mediating cognitive processes of learning and memory [5] and this is interesting because it may explain the fact that the development of fatigue is associated with impairment cognitive performance in these patients.

This assumption has led many studies to support the hypothesis of CNS cholinergic involvement in MG [3], [6], which suggests that MG has central cholinergic deficits manifested by cognitive dysfunction [6]. Also, various investigations have proposed that the CNS and the non-striated muscles might be affected in MG. Moreover, reports of REM sleep reduction [7], memory dysfunction [6], and detection of AChRs-antibodies in the cerebrospinal fluid confirmed the hypothesis of CNS involvement in MG [8]. Thus, if CNS involvement does occur in MG, it might reasonably be expected to manifest itself in certain abnormalities of cognition in view of the evidence of the role of cholinergic transmission in attention and memory.

Then again, evidence for CNS involvement in MG is remarkably unconvincing [9]. According to Marra et al. [10], impairments of attention, memory, and control tasks in MG are related to general visual motor slowness and to the concomitant presence of other diseases and not to cholinergic involvement in MG. Moreover, Sitek et al. [11] suggested that impaired performance on some cognitive measures in MG should be interpreted as an effect of muscle fatigability rather than CNS involvement. Thus, it remains unclear as to whether MG affects the CNS.

However, it has been emphasized by Paul et al. [12] the need for more studies with sufficient methodological approaches, which will investigate the cholinergic hypothesis in MG. Thus, the aim of this study was to examine with established neurophysiological and neuropsychological methods, such as pupillometry and concomitantly with cognitive test and Hamilton Depression Rating Scale (HAM-D), the hypothesis of CNS cholinergic involvement in patients with MG.

## 2 Material and methods

### 2.1 Subjects

Thirty-two (32) patients with a mean age 51.1 ± 17.2 years and diagnosed with MG volunteered to participate in this study, while thirty- three (33) healthy subjects with a mean age 50.2 ± 14.8 served as controls. The inclusion criteria for the present study were a definitive MG diagnosis and clinical condition not worse than MGFA grade II during the last year before participation. The mean time from the diagnosis until the beginning of the study was 2.9 ± 0.2 years. Specifically, their diagnosis was based on the one hand on the clinical symptoms (fatigue during the day, etc.), and on the other hand, on the laboratory findings (the presence of positive antibodies for the AchR, positive response at the repeated stimuli test). Apart from these, MG patients showed an improvement of muscle strength following the intake of edrophonium chloride and reacted positively to Mestinon.

All patients were free of any other neurological, ophthalmological, physical or mental disease and their visual acuity, corrected or not, was 20/20. Moreover, they had symmetrical pupils and no past history of ocular operations or diseases affecting the pupil and were not being treated at that time with anticholinergics, steroids, sympathomimetics, β-blockers or other agents affecting the pupil light reflex (PLR). Also, the basic precondition for participation was a mini mental score ≥ 22. All measurements were performed between 09.00 am and 10.00 am, after the participants had had a full 8-h sleep.

The whole study was conducted in the A Neurology Clinic of AHEPA University Hospital of Aristotle University of Thessaloniki. All participants were informed about the study procedure and provided written informed consent and all the experiments were approved by the Ethical Committee of the AHEPA University Hospital based on the Helsinki Declaration. At the entry of the study all participants underwent a neuropsychological evaluation with pupillometry, Wechsler Memory Scale (WMS) and HAM-D. All participants were assessed on a single occasion only.

### 2.2 Pupillometry

Pupillary measurements were taken with a monocular and fully automated system that includes the following:

1) A CCD high-speed digital camera, which is capable of taking a maximum of 262 frames per second. The actual speed of the camera is controlled by the software developed for this purpose. Because of the corneal curvature and in order to avoid errors due to optical distortion, the camera is set normal to the axis of the eye and at a distance of 30 cm away, so that the image of the pupil is symmetrical.
2) A computer and the associated sampling cards
3) Two independent light sources: a) an infrared light source which illuminates the face of the subject, consisting of an array of 32 LED with a maximum intensity of 820 nm wavelength and is switched on permanently throughout the measurement and b) a clinical photic stimulator (SLE) made by Biologic Systems Corporation U.K., which activates a diffuse flashlight of 20 ms duration and 24.6 cd/m^2^ intensity
4) A traversing mechanism with a fixed camera on a mechanism which can move in the three x–y–z directions with also, a capability to rotate the camera on both the x–y and the x–z planes
5) An image processing system, which calculates the parameters of the PLR in real time. The recordings of these parameters cease automatically after 3.5 s from the application of flash-light.

The accuracy of measurements depends on the number of pixels covered by the pupil, which depends upon the optics of the system. In the present case, the pupil covers about 120 × 120 pixels, so the accuracy of measurement of distance is ± 0.62%. This leads to a spatial resolution in both the x and y directions of 0.015 mm. The temporal resolution is 2 ms [3].

Participants remained for 2 min in darkness, and 5 flashes were administered afterwards (inter-stimulus interval was 30 s). Stimulus duration was 20 ms, and the luminance 24.6 cd/m^2^. Each eye was tested separately. The flash was placed at a distance of 30 cm from the eye. Both healthy and myasthenic participants performed the experiment with proper cooperation.

The following pupillometric parameters were measured:

1) Latency for the onset of constriction (T1), which is an index of sympatho-vagal balance, since it is involved in the second segment of the V-shaped pupillometric response [13]. According to Bergamin et al. [14] T1 is defined at the time of maximum constriction acceleration;
2) Maximum constriction velocity (VCmax), which is a sensitive index of Parasympathetic Nervous System (PNS) activity [13], [15].
3) Maximum constriction acceleration (ACmax), which is modulated by PNS activity [13], [15].

### 2.3 Cognitive tests and HAM-D

#### 2.3.1 Wechsler Memory Scale (WMS)

The Greek version of the WMS [16] was used to evaluate the patients' different memory functions. WMS is an established neuropsychological test, which evaluates patient' memory functions in relation to other cognitive abilities and it is the most frequently used standardized measure of memory function.

#### 2.3.2 Hamilton Depression Rating Scale (HAM-D)

The severity of depression was assessed with the 17-item HDRS, which is one of the most widely used observer-rated instruments to assess the severity of depressive symptoms. The Greek version of the HAM-D was used. Eight items are scored from 0 to 2 and nine items are scored from 0 to 4. A cut-off point of 17 is often used to ensure a degree of depression severity [17].

### 2.4 Statistical analysis

Data were analysed with Statistical Package for Social Sciences (SPSS, Chicago, Illinois, USA), version 20.0 software for Windows. The Kolmogorov–Smirnov test was used to examine the normality of the distribution. Changes of variables within the groups at baseline and the end of the study were evaluated by a One-Way ANOVA with group being the independent variable. The significance level was p < 0.05. Finally, the Pearson correlation was calculated between each pair of pupillometric parameters for each patient group and for the total group of the healthy people. Moreover, the correlation of each pupillometric parameter with age was also estimated. Curve analysis (ROC) was performed in order to illustrate the classification and discrimination accuracy of the pupillary light reflexes. The area under the curve (AUC) of the ROC curves was estimated and used as the index of classification accuracy, where a variable with an AUC = 1 indicates excellent discrimination ability into the myasthenic or healthy group while a variable with an AUC near 0.5 indicates poor discrimination ability into the two groups.

## 3 Results

**Fig. 1:**
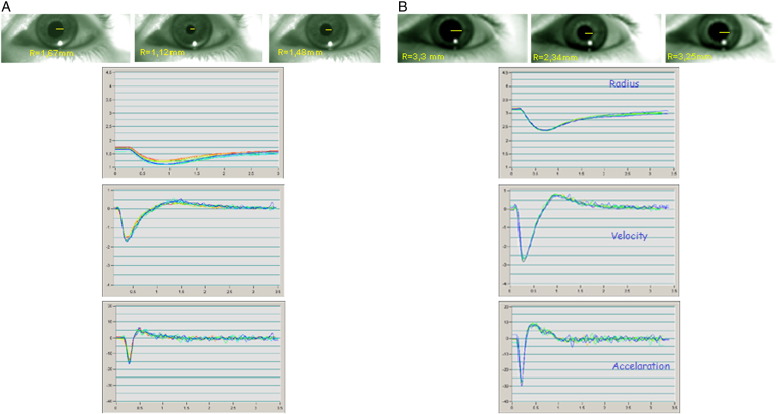
Typical pupil light reflex curve of a myasthenic patient (A) and a non-myasthenic male (B) of matching age and gender.

**Fig. 2:**
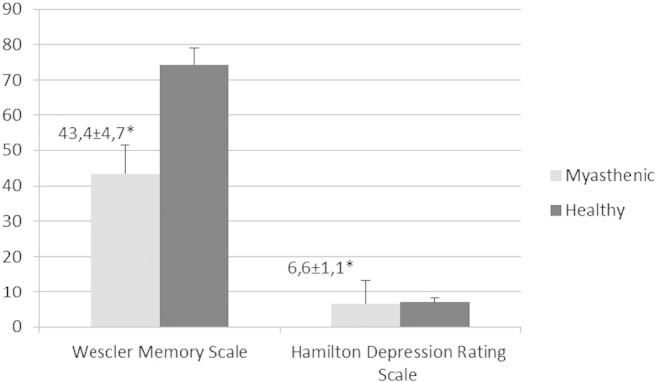
Cognitive test and Hamilton Depression Rating Scale results.

**Fig. 3:**
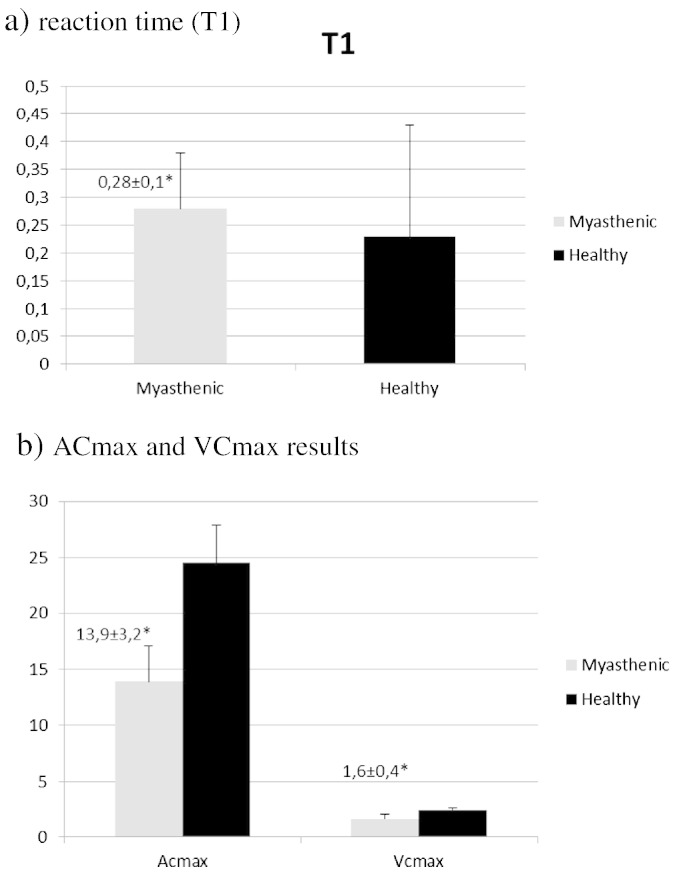
Pupillometry results: a reaction time(T1).

Descriptive statistics of all variables are presented in Table 1. MG patients and controls were comparable for age, educational level, and gender. No significant differences were found for age and HAM-D between the two groups. Also, patients with MG had abnormal pupillary function as compared to healthy subjects. Specifically, the mean value of T1 was found significantly increased by 21.7% (p < 0.05) in MG patients as compared to healthy subjects. Conversely, the mean scores of VCmax and ACmax were significantly decreased in MG patients by 33.3% (p < 0.05) and by 43.5% (p < 0.05), respectively, as compared to healthy subjects. Patients with MG had decreased score in WMS by 41.6% (p < 0.05) compared with healthy subjects. Moreover, WMS score was correlated with the pupillometric indexes of ACmax (r = 0.602, p < 0.05) and VCmax (r = 0.578, p < 0.05). In Fig. 1 the PRL is presented for a non-myasthenic male (a) and a myasthenic patient (b). See Fig. 2, Fig. 3

ACmax and VCmax were the best predictors in discriminating a subject as myasthenic or healthy with perfect classification ability (AUC = 1). Also, T1 had perfect classification ability (AUC = 0.945).

## 4 Discussion

In the present investigation, the hypothesis concerning a central cholinergic receptor's deficit in MG patients was studied using neurophysiological and neuropsychological methods. The results of this study demonstrated that MG patients exhibited a decreased cholinergic activity, indicating that MG affects the cholinergic system and leads to reduced cognitive performance.

Pupillometry is a valid and low-cost method for the evaluation of ANS activity [18], [19], [20]. The pupil is controlled by two kinds of muscles whose innervations are different: the iris sphincter is innervated by the PNS, and the iris dilator, by the Sympathetic Nervous System (SNS). Therefore, pupillometry has a potential to allow independent evaluation of both types of ANS activity [18], [20]. Accordingly, changes in the pupil size can be used to assess the PNS and SNS neurotransmitters' function, ACh and noradrenaline, respectively, within the CNS [18], [20].

ACmax and VCmax are considered to be extremely sensitive indicators of cholinergic activity [3]. Yamazi et al. [13] had divided the characteristic V-shaped response of the pupillary light reflex into three segments: the first is due exclusively to PNS excitation, the second is controlled by both SNS and PSN and the latter reflects only SNS activity. Hence, there is evidence that the parameters involved in the first segment, such as ACmax and VCmax, are sensitive indicators of cholinergic activity. According to our data, there was a significant decrease in VCmax and ACmax in MG patients, leading to the hypothesis that there is a central cholinergic deficit in MG. Our results are in accordance with other studies which support that MG affects the cholinergic system [6].

The significant decrease of the acceleration and the velocity ascertains the cholinergic deficit, since ACh is considered the main neurotransmitter in the pupil movement. It could be supported that ACh is the fuel of this movement and its deficit results in the deceleration of the motion of the pupil in response to light stimulus. Decreased ACmax and VCmax in patients with MG have been previously reported in a study by Tsiptsios et al. [3], supporting the CNS cholinergic deficit hypothesis. None of the 32 patients who underwent pupillometry complained for disturbances in adjustment to light or to darkness. Besides, all patients underwent the ophthalmologic examination test with success. This means that the decrease of ACh does not influence the motion of the pupil to the extent that it could cause difficulties of adjustment for the patient or pathologic findings in the clinical ophthalmologic examination. The hypothesis concerning CNS involvement in MG is based on concurrent incidence of MG and multiple sclerosis, a prototypic autoimmune disease of the CNS, implicating a common pathogenic mechanism for the two diseases [21], [22], [23]. Thus, according to the aforementioned, the hypothesis concerning a central cholinergic receptor's deficit in MG is supported and the use of VCmax and ACmax as the most sensitive indicators of this deficit is confirmed.

Moreover, MG patients significantly displayed more difficulties in cognitive abilities as revealed by their lower performance at the WMS. These patients may have a degree of difficulty to memory functions in relation to other cognitive abilities. This indicates the existence of some cognitive dysfunction in MG patients, which has also been reported in other studies [24], [25]. In addition, the HAM-D was used to assess the severity of depressive symptoms and to exclude this bias, because typical depression in older adults is usually associated with mild reductions in cognitive functioning.

However, this is the first study that has dealt with the PLR to single Flash Stimuli and cognitive tests in MG. The fact that cholinergic deficit was observed in MG patients combined with the important role that nicotinic AChRs in the hippocampus, hypothalamus and cerebral cortex play in higher cognitive functions, supports the CNS involvement hypothesis.

## 5 Conclusions

In conclusion, this is the first investigation that has dealt with the pupillary light reflex to single Flash Stimuli and the concomitant use of the WMS in MG. The results proposed that the Central Cholinergic System and/or the iris sphincter smooth muscle might be affected. Furthermore, cognitive dysfunction was observed in patients with MG. Finally, it is suggested that pupillometry used concomitantly with the WMS contributes to the elucidation and understanding of the psychophysiological bases of MG.

## Transparency document

Transparency document.

## Figures and Tables

**Table 1:** Descriptive statistics and comparison between myasthenic patients and healthy subjects for all variables.

Variables	MG patients (n = 32)	Healthy subjects (n = 33)	MG vs. healthy p-Value⁎
Mean ± s.d.	Mean ± s.d.	p < 0.05
*Demographic characteristics*			
Males/females	15/17	16/17	–
Age	51.1 ± 17.2	50.2 ± 14.8	0.086
Educational level (yrs)	7.9 ± 3.2	8.1 ± 4.1	0.16
Disease duration (yrs)	2.9 ± 0.2	–	–

*Cognitive test and Hamilton Depression Rating Scale*			
Wechsler Memory Scale	43.4 ± 4.7	74.4 ± 8.0	< 0.001*
Hamilton Depression Rating Scale	6.6 ± 1.1	7.0 ± 6.8	0.091

*Pupillometric parameters*			
T1	0.28 ± 0.1	0.23 ± 0.2	< 0.001*
ACmax	13.9 ± 3.2	24.6 ± 3.3	< 0.001*
VCmax	1.6 ± 0.4	2.4 ± 0.2	< 0.001*

T1: Latency for the onset of constriction, ACmax: Maximum constriction acceleration, VCmax: Maximum constriction velocity.

⁎ p < 0.05 considered significant.
